# Vitamin D Promotes Trophoblast Cell Induced Separation of Vascular Smooth Muscle Cells in Vascular Remodeling *via* Induction of G-CSF

**DOI:** 10.3389/fcell.2020.601043

**Published:** 2020-12-22

**Authors:** Joy Yue Zhang, Peihuang Wu, Danyang Chen, Fen Ning, Qinsheng Lu, Xiu Qiu, Martin Hewison, Jennifer A. Tamblyn, Mark D. Kilby, Gendie E. Lash

**Affiliations:** ^1^Division of Uterine Vascular Biology, Guangzhou Institute of Pediatrics, Guangzhou Women and Children’s Medical Center, Guangzhou Medical University, Guangzhou, China; ^2^Born in Guangzhou Cohort, Guangzhou Women and Children’s Medical Center, Guangzhou Medical University, Guangzhou, China; ^3^College of Medical and Dental Sciences, Institute of Metabolism and Systems Research, University of Birmingham, Birmingham, United Kingdom; ^4^Fetal Medicine Centre, Birmingham Women’s and Children’s NHS Foundation Trust, Birmingham, United Kingdom

**Keywords:** vitamin D, spiral artery remodeling, extravillous trophoblast cells, G-CSF, VSMC phenotypic switching

## Abstract

Vitamin D deficiency is associated with complications of pregnancy such as pre-eclampsia, fetal growth restriction, and miscarriage, all of which are also associated with incomplete spiral artery (SpA) remodeling. We have previously shown that both uterine natural killer (uNK) cells and extravillous trophoblast cells (EVT) are required for successful SpA remodeling, but whether their activity in this process is modulated by vitamin D is not known. In the current study, we use a previously described chorionic plate artery (CPA) *ex vivo* model of vascular remodeling to determine the effects of 1,25(OH)_2_D treated uNK cell, placental explant (PEx), and uNK/PEx conditioned medium (CM) on vascular smooth muscle cell (VSMC) disorganization and phenotypic switching. Significant results were followed up in VSMCs *in vitro*. We demonstrate that 1,25(OH)_2_D can enhance the ability of PEx to induce SpA remodeling, *via* a mechanism associated with increased secretion of granulocyte-colony stimulating factor (G-CSF). G-CSF appears able to increase VSMC disorganization and phenotypic switching in both an *ex vivo* vascular model and *in vitro* VSMC cultures. The clinical relevance of these findings are still to be determined. G-CSF may have differential effects depending on dose and vascular bed, and vitamin D may play a role in potentiating these actions. G-CSF may be an interesting potential therapeutic target for facilitating physiological vascular remodeling for the prevention of adverse obstetric outcomes.

## Introduction

Vitamin D is a secosteroid classically recognized for its key role in bone metabolism and calcium homoeostasis (Haussler et al., 2008). Many important “non-classical” extra-skeletal functions of vitamin D have been described, including anti-proliferative, pro-differentiative, and potent immunomodulatory actions (Hewison et al., 2007; Hewison, 2010). Non-classical actions of vitamin D are associated with local metabolism of precursor 25-hydroxy vitamin D (25OHD), with the resulting active 1,25(OH)_2_D signaling *via* endogenous vitamin D receptors (VDRs) (Hewison et al., 2007; Hewison, 2010). Early in human pregnancy, the maternal decidua and fetal trophoblast cells express both VDR and 1α-hydroxylase (CYP27B1), an enzyme that catalyzes conversion of 25OHD to 1,25(OH)_2_D (Gray et al., 1979; Weisman et al., 1979). By contrast, the vitamin D catabolic enzyme, 24-hydroxylase (CYP24A1), has reduced expression in placental/decidual tissues across gestation (Zehnder et al., 2002; Evans et al., 2004). In this way, the placental/decidual tissues have the potential to generate significant amounts of 1,25(OH)_2_D without appreciable catabolic inactivation, allowing potential paracrine or autocrine action (Tamblyn et al., 2017).

The health implications of vitamin D deficiency (definition: deficiency as 25OHD < 30 nM and insufficiency as 25OHD < 50 nM) are far reaching. Classically, severe vitamin D deficiency is recognized for its negative effect on bone mineralization, as manifested by rickets in children and osteomalacia in adults (Holick, 2007; Holick and Chen, 2008). In more recent studies, low serum levels of 25OHD have been associated with common cancers, allergic disorders, infections, autoimmune disorders, and cardiovascular disease (Hypponen et al., 2001; Cannell et al., 2006; Munger et al., 2006; Grant and Mohr, 2009; Brehm et al., 2010; Zittermann et al., 2011). In addition, vitamin D deficiency is linked and associated with a number of complications of pregnancy including miscarriage (previous loss before 24 weeks), pre-eclampsia, gestational diabetes, preterm birth, and pregnancies complicated by small for gestational age fetus (Bodnar et al., 2007, 2010, 2015; Wei et al., 2013; Gernand et al., 2014). All of which are also associated with deficiencies in maternal uterine adaptation to pregnancy (Pijnenborg et al., 2006). We recently demonstrated that reduced local levels of active vitamin D at the maternal-fetal interface, through altered expression of vitamin D catabolizing enzymes, is associated with spontaneous first trimester miscarriage (Hou et al., 2020). While it has been shown that active vitamin D plays a role in regulation of extravillous trophoblast (EVT) invasion its role in spiral artery (SpA) remodeling is not clear (Chan et al., 2015).

Spiral arteries are blood vessels of the uterus arising from the uterine artery which extend from within the myometrium into the decidua in pregnancy and deliver maternal blood to the placental which extracts oxygen and nutrients for maintenance of both the placenta itself and growth and development of the fetus (Pijnenborg et al., 2006). During the process of SpA remodeling the vessels lose their musculoelastic wall which is replaced by a dense fibrinoid material incorporating intramural EVT, *via* a process involving separation and de-differentiation (phenotypic switching) of the vascular smooth muscle cells (VSMCs), and their eventual loss from the vessel wall *via* migration into the surrounding stroma and apoptosis (Pijnenborg et al., 2006; Bulmer et al., 2012; Robson et al., 2019). Failure of the remodeling process is associated with a number of complications of pregnancy including pre-eclampsia, fetal growth restriction, and miscarriage (Khong et al., 1986; Pijnenborg et al., 1991; Ball et al., 2006). The molecular triggers of these intricate processes are not well understood, though roles for angiogenic growth factors and cytokines have been proposed. Histological evidence suggests that while EVT are critical for complete SpA remodeling, the process starts in the absence of EVT and is likely initiated by decidual leucocytes (Craven et al., 1998; Kam et al., 1999; Smith et al., 2009; Lash et al., 2016). We have previously demonstrated that uterine natural killer (uNK) cells can initiate SpA remodeling by inducing separation and rounding up of the VSMCs *via* a mechanism associated with secretion of angiogenic growth factors (Robson et al., 2012). However, EVT cells were not able to induce such changes, nor was the conditioned medium from uNK cells co-cultured with EVT (Robson et al., 2012). In addition, uNK cells and EVT were able to induce VSMC phenotypic switching from a contractile to a more synthetic phenotype, *via* a mechanism also associated with secretion of angiogenic growth factors (Robson et al., 2019). Other studies have suggested that EVT induce VSMC apoptosis within the vessel wall to facilitate their loss, however, little evidence of VSMC apoptosis is seen histologically unless the VSMC has already migrated away from the vessel wall (Harris et al., 2006; Keogh et al., 2007; Bulmer et al., 2012). *In vitro* studies have suggested that EVT may induce VSMC phenotypic switching and increase VSMC motility *via* a range of mechanisms including CXCL10, EVT secreted exosomes and long non-coding RNA MEG3 (Wallace et al., 2013; Salomon et al., 2014; Tao et al., 2019). Previous studies had also shown that uNK cell and EVT co-culture reduced secretion of various cytokines and angiogenic growth factors (Lash et al., 2011). Vitamin D is also able to alter cytokine secretion by uNK cells (Evans et al., 2006).

Vascular smooth muscle cells show a remarkable plasticity and do not become terminally differentiated but can switch phenotype from synthetic to contractile and vice versa depending on the environmental cues (Owens, 2007). This phenotypic switching can be monitored by investigation of a number of VSMC markers including acquisition of VSMC contractile proteins [α-smooth muscle actin (SMA), smoothelin, calponin, H-caldesmon, and myosin heavy chain etc.] and loss of osteopontin as they become more contractile, and the reverse as they switch back to a synthetic state (Owens, 2007).

Vitamin D deficiency is associated with the same complications of pregnancy as incomplete SpA remodeling. We therefore hypothesized that vitamin D plays a role in SpA remodeling by enhancing the ability of uNK cells and/or EVT to facilitate the initial stages of this process including VSMC disorganization and VSMC phenotypic switching.

## Materials and Methods

### Subjects and Sample Collections

Human placenta and decidua samples were obtained from women undergoing elective surgical termination of apparently uncomplicated pregnancies between 5–7 weeks gestation (determined by ultrasound) in Guangzhou Women and Children’s Medical Center (GWCMC). Placental and decidua samples were separated and washed thoroughly with sterile saline. Samples for immunohistochemistry (IHC) were fixed with 10% (v/v) formalin and embedded in paraffin (*n* = 7). Samples for isolation of uNK cells (decidua) or placental explants (PEx, placenta) were processed immediately as described below (*n* = 10 decidua and placenta). Chorionic plate artery (CPA) segments, which were used as an *in vitro* model to analyze the effect of uNK cell and PEx products on VSMC morphology, were obtained from normal term placenta delivered by elective cesarean section. The study was approved by the Ethical Review Board of GWCMC. According to local guidelines it was not necessary to obtain written informed consent for the use of these tissues.

### Isolation and Culture of CD56+ uNK Cells, PEx, and uNK Cell/PEx Co-cultures

Paired decidua and placenta were taken from the same patient after termination of pregnancy for isolation of uNK cells and PEx, respectively.

uNK cell-enriched isolates were prepared by enzymatic disaggregation and positive immunomagnetic selection (#130-092-657, Miltenyi Biotech, Bisley, United Kingdom) as described previously (Lash et al., 2006). To prepare uNK cell-conditioned medium (uNK-CM), cells were cultured at 1 × 10^6^ cells/ml in RPMI1640 complete medium (RPMI containing 10% (v/v) fetal calf serum, 1% (v/v) penicillin/streptomycin, 1% (v/v) amphotericin; all from Gibco, life Technologies, United States) containing 1,25(OH)_2_D (0, 1, and 10 nM; BML-DM200, ENZO, Switzerland) or 25OHD (0, 10, and 100 nM; BML-DM100, ENZO, Switzerland). Stock solutions (10 and 100 mM respectively) of 1,25(OH)_2_D and 25OHD were prepared in 100% ethanol and further dilutions prepared in phosphate buffered saline (PBS), controls included 0.001% ethanol as per the highest concentration of each agent. After 24 h the uNK-CM was removed, centrifuged and stored at −80°C. Cell purity and viability were tested routinely by immunocytochemical staining of cell smears and trypan blue exclusion, respectively; both were consistently >95%.

PEx were isolated as previously described (Lash et al., 2005) and approximately 10 mg tissue cultured in DMEM/F12 (Gibco-C11330500BT, Life Technologies, United States) complete medium containing 1,25(OH)_2_D (0, 1, and 10 nM) or 25OHD (0, 10, and 100 nM) in a 24 well plate coated with growth factor reduced Matrigel (BD-354230, BD Biosciences, Oxford, United Kingdom). Conditioned medium was harvested (PEx-CM) after 24 h, centrifuged and stored at −80°C.

For direct co-culture of uNK cells and PEx, 10 mg PEx were cultured in DMEM/F12 complete medium in a 24 well plate overnight prior to addition of 1 × 10^6^ uNK cells (from the same patient) in DMEM/F12 containing 1,25(OH)_2_D (0, 1, and 10 nM) or 25OHD (0, 10, and 100 nM) to a total volume of 1 ml (Lash et al., 2011; Robson et al., 2012). For indirect co-culture the uNK cells were added to a transwell insert with a 0.4 μm filter (#3413, Corning, United States) but all other conditions were the same as for the direct co-culture. After 24 h co-culture, the conditioned medium (uNK/PEx-dir-CM or uNK/PEx-indir-CM) was removed, centrifuged and stored at −80°C. Viability of co-cultures was assessed after 24 h of culture and was consistently >95%.

### Isolation and Culture of Chorionic Plate Arteries

Intact CPA segments (5 mm length) (Robson et al., 2012, 2019; Pitman et al., 2013; Lash et al., 2016) were dissected from normal term placenta and cultured at 37°C in 5% CO2 in: (A) conditioned medium: 20% (v/v) uNK-CM, PEx-CM, uNK/PEx-dir-CM, or uNK/PEx-indir-CM all with or without 1,25(OH)_2_D (0, 1, and 10 nM) or 25OHD (0, 10, and 100 nM) (*n* = 5 each group); (B) granulocyte-colony stimulating factor (G-CSF) (0, 20, 50, and 100 pg/ml; AF-300-23, PeproTech, United States) (*n* = 6); (C) 20% (v/v) PEx-CM and anti-G-CSF (50 ng/ml, Ab-9691, AbCam, Cambridgeshire, United Kingdom) (*n* = 6); or (D) RPMI1640 complete medium control.

The medium was completely replaced every 48 h. CPAs were harvested after 120 h, fixed in 10% (v/v) neutral buffered formalin for 24 h and embedded in paraffin wax. 3 μm serial sections were stained with hematoxylin and eosin (H & E).

### Luminex

Cytokine, chemokine, and angiogenic growth factor levels were tested uNK-CM, PEx-CM, uNK/PEx-dir-CM and uNK/PEx-indir-CM all with or without 1,25(OH)_2_D (0, 1, and 10 nM) or 25OHD (0, 10, and 100 nM) (*n* = 5 each group) by Luminex multiplex assay according to the manufacturer’s instructions (Millipore). The analytes tested were EGF, IFN-γ, IL-1RA, IL-1β, IL-4, IL-6, MDC, PDGF-AA, PDGF-BB, VEGF-A, FGF-2, MCP-1, MCP-3, MIP-1α, MIP-1β, RANTES, TNF-α, PLGF, IP-10, G-CSF, GM-CSF, GRO-α, and Ang-2.

### Vascular Smooth Muscle Cell (VSMC) Culture

Human Umbilical Artery Smooth Muscle Cells (HUASMC) were obtained from ScienCell (Cat. No. 8030, ScienCell, United States) and cultured in smooth muscle cell medium (SMCM; #1101, ScienCell, United States) supplemented with 2% (v/v) fetal bovine serum (FBS; #0010), 1% (v/v) penicillin/streptomycin (P/S; #0503), 1% (v/v) smooth muscle cell growth supplement (SMCGS; #1152) maintained in a standard tissue culture incubator at 37°C, 5% CO_2_ in air. VSMCs were used between passages three and six.

### Proliferation and Invasion Assessment Using the Real-Time Cell Analysis xCELLigence System

The RTCA utilizes specialized plates that contain interdigitated electrodes which can be used to determine cell proliferation, migration, and invasion in real time (Xi et al., 2011; Roshan Moniri et al., 2015). For all measurements the RTCA chamber was housed within a cell culture incubator at 37°C and 5% CO_2_ in air.

For proliferation assays 5 × 10^3^ VSMCs in 150 μl complete SMCM medium containing 0, 0.1, or 3 ng/ml G-CSF were added to duplicate wells of an E-Plate. Recordings were taken every 15 min for a total of 48 h (*n* = 3).

For invasion assays the upper chamber of a CIM-16-Plate was coated with 20 μl growth factor reduced Matrigel diluted 1:30 with serum free SMCM medium and allowed to set for 4 h at 37°C in a 5% CO_2_ in air cell culture incubator. Complete SMCM medium was added to the lower chamber prior to attachment of the upper chamber and addition of 2 × 10^4^ VSMCs in serum free SMCM medium containing G-CSF (0, 0.1, and 3 ng/ml). Recordings were taken every 15 min for a total of 48 h (*n* = 3).

### Immunohistochemistry

Human CPA segments, decidua, and placenta samples were collected and fixed as described above. 4 μm serial sections were cut, deparaffinized in xylene and reydrated in 95% (v/v), 85% (v/v), 75% (v/v) ethanol, and dH2O (5 min each step). According to the antibody datasheets antigen retrieval was performed in either citrate buffer (pH6.) or EDTA buffer (pH8.0) and heat. After samples were cooled to room temperature sections were incubated in 3% (v/v) H_2_O_2_ for 10 min. Samples were stained with specific antibodies overnight at 4°C after 30 min blocking with 10% (v/v) goat serum. Antibodies to human alpha smooth muscle Actin (αSMA, ab32575), osteopontin (OPN, ab69498), anti-G-CSF (ab204989), and anti-G-CSF Receptor (ab126167) were purchased from Abcam. All antibodies were used in a 1:200 dilution for IHC, while negative control was performed by replacing the primary antibody with PBS or the appropriate IgG control. After washing, the slides were incubated with DAKO Real^TM^ EnVision^TM^ Detection System, Peroxidase/DAB, Rabbit/Mouse (Cat. K5007, DAKO, Denmark) and detected by 3,3-diaminobenzadine (DAB). Analysis of each section using a modified “quickscore” method taking into account both percentage of cells stained (1 = 0–25%, 2 > 25–50%, 3 > 50–75%, and 4 > 75–100%) and the staining intensity for each percentage (0 = negative, 1 = weak, 2 = moderate, and 3 = strong). The percentage and intensity scores were then multiplied and summed to give a possible score range of 0–12 (Schiessl et al., 2009).

### Immunofluorescence of VSMC Specific Biomarkers

The VSMCs were cultured up to 80% confluence in an 8-well Nunc Lab-Tek Chamber Slide (#154534, ThermoFisher, United States), medium removed, rinsed with PBS and fixed with 4% (v/v) paraformaldehyde for 30 min. The cells were permeabilized with 0.5% (v/v) Triton-X100 for 15 min and then blocked in 10% (v/v) goat serum for 30 min. The slides were incubated overnight at 4°C with primary antibodies to αSMA (ab32575, 1:200), OPN (ab69498, 1:200), and KLF4 (ab215036, 1:200). After washing, the slides were incubated with goat anti-rabbit IgG (H + L) superclonal^TM^ secondary antibody Alexa Fluor 555 (1:1,000 dilution) or goat anti-mouse IgG (H + L) superclonal^TM^ secondary antibody Alexa Fluor 488 (1:1,000 dilution) for 1 h and then DAPI (1:1,000 dilution) for 10 min. Images were captured using a Leica fluorescent microscope using a 40X objective and staining intensity analyzed using Image J software. At least three images were analyzed per condition over three separate experiments.

### Immunofluorescence of F-Actin (the Actin Cytoskeleton)

The VSMCs were cultured up to 80% confluence in an 8-well Nunc Lab-Tek Chamber Slide (#154534, ThermoFisher, United States), medium removed, rinsed with PBS and fixed with 4% (v/v) paraformaldehyde for 30 min. The cells were permeabilized with 0.5% (v/v) Triton-X100 for 15 min and then blocked in 10% (v/v) goat serum for 30 min. The slides were incubated with Texas Red-X Phalloidin (T7471, ThermoFisher, United States) for 1 h prior to DAPI (1:1,000 dilution) staining at room temperature. For each treatment, 5 images were taken randomly within each chamber *via* a Bio-Rad ZOE Fluorescent Cell Imager using a 20X objective. F-actin structures in individual cells were observed and categorized into three types (Park et al., 2019). Quantification of each type of F-actin structure was analyzed using Image J software (Park et al., 2019). Briefly, stress fibers, where F-actin stress fibers were observed both in the nucleus and cytoplasm; transition state fibers, where F-actin stress fibers were observed only in the cytoplasm; cortical structure fibers, where F-actin fibers were observed near the cellular membrane as cortical structures.

### Western Blot Analysis

Control and G-CSF (3 ng/ml) treated VSMCs were washed twice with cold phosphate-buffer saline (PBS) and then protein extracted with RIPA buffer (Beyotime, Cat: P0013B, Shanghai, China) containing PMSF (Beyotime, Cat:ST506) and protease inhibitor cocktail (CWBIO, Cat: CW2383S, Jiangsu, China). Cell lysates were centrifuged at 12,000 *g* at 4°C for 30 min and supernatant collected and stored at −80°C until required for analysis. Protein concentrations were determined by BCA protein assay kit (Beyotime, Cat: P0009). 20 μg total protein was mixed with 5X loading buffer and denatured at 95°C for 8 min before being separated on SDS-PAGE, and electro-transferred onto polyvinylidene difluoride (PVDF) membranes (Millipore, Cat: IPVH00010, Billerica, MA, United States). Membranes were blocked with 5% (w/v) skimmed milk for 2 h, and incubated with target antibodies on a shaking bed overnight at 4°C. Primary antibodies used in this study were: Anti-SMA, Cat: ab32575, rabbit, 1:1,000; anti-OPN, Cat: ab69498, mouse, 1:1,000; anti-GAPDH, Cat: ab181602, rabbit, 1:10,000 (AbCam, Cambridge, United Kingdom). After removing the primary antibodies, membranes were washed with TBST 3 × 10 min. The membranes were then incubated with HRP-conjugated secondary antibodies on a shaking bed for 2 h at room temperature. After washing three times with TBST, chemiluminescent substrate was used to develop and signals were captured by ChemiDoc XRS+ imaging system (Bio-Rad, CA, United States). Data were analyzed by image J software (NIH, United States). Each experiment was repeated three times.

### Statistical Analysis

Data are presented as mean ± SD. Statistical significance was determined using Statview by one-way ANOVA with Fisher’s *post hoc* test or Student’s *t*-test as appropriate. A *P* value of <0.05 was considered significant.

## Results

### Vitamin D Enhances the Ability of Placental Explants to Induce VSMC Disorganization in the CPA Model

**FIGURE 1:**
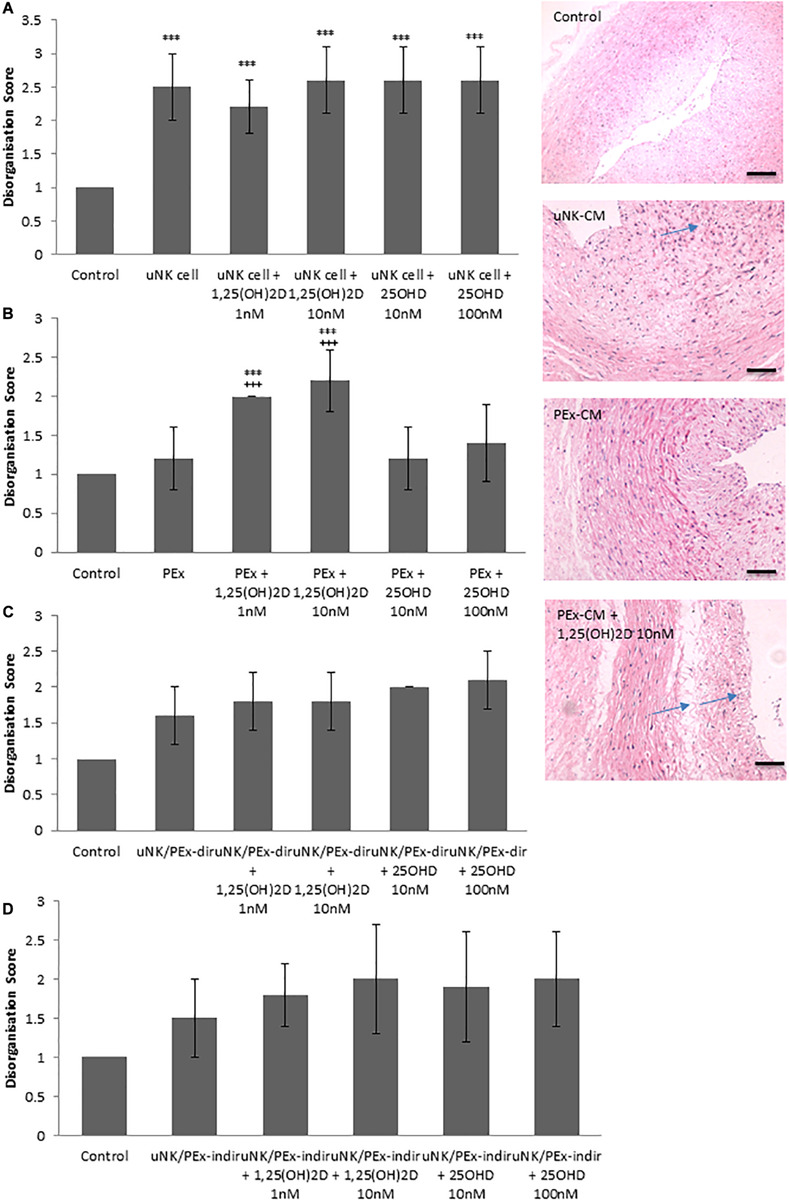
Effect of vitamin D on uNK cells and PEx ability to induce VSMC disorganization in a CPA model of spiral artery remodeling. **(A)** uNK cell CM induced VSMC disorganization, but vitamin D had no further effect. **(B)** PEx CM had no effect on VSMC disorganization, but treatment with vitamin D induced the ability of PEx CM to alter VSMC organization. **(C,D)** uNK cell and PEx in co-culture, either direct or indirect co-culture had no effect on VSMC disorganization, which was also not altered by treatment with vitamin D. *N* = 5 each condition; ***P < 0.0001 vs. control; +++*P* < 0.0001 vs. PEx alone. Arrows denote examples of disorganization. Scale bars = 50 μm.

To determine the effect of vitamin D treated uNK cell, PEx or co-culture CM on VSMC organization a CPA model of vascular remodeling was used (Robson et al., 2012). As previously shown uNK cell CM induced VSMC disorganization (*P* < 0.0001) (Robson et al., 2012), however, further treatment with either 1,25(OH)_2_D or 25OHD did not alter uNK cell mediated VSMC disorganization (Figure 1A). PEx CM also had no effect on VSMC disorganization in the CPA model (Robson et al., 2012). However, 1,25(OH)_2_D (1 nM *P* = 0.0001 and 10 nM *P* < 0.0001) treated PEx CM induced VSMC disorganization compared to control and PEx CM alone (Figure 1B). CM from uNK cells and PEx in co-culture, either in direct contact or separated by a transwell filter, did not induce VSMC disorganization in accordance with our previous findings, and treatment with 1,25(OH)_2_D or 25OHD did not alter the ability of these co-cultures to induce changes in the CPA model (Figures 1C,D). Both 1,25(OH)_2_D (10 nM) and 25OHD (100 nM) induced VSMC disorganization in the CPA model (data not shown). But since treatment of PEx with 1 nM 1,25(OH)_2_D treated PEx induced disorganization we conclude that this is a specific PEx mediated response and not due to residual vitamin D in the conditioned medium.

### PEx Increased Secretion of G-CSF in Response to 1,25(OH)_2_D

**FIGURE 2:**
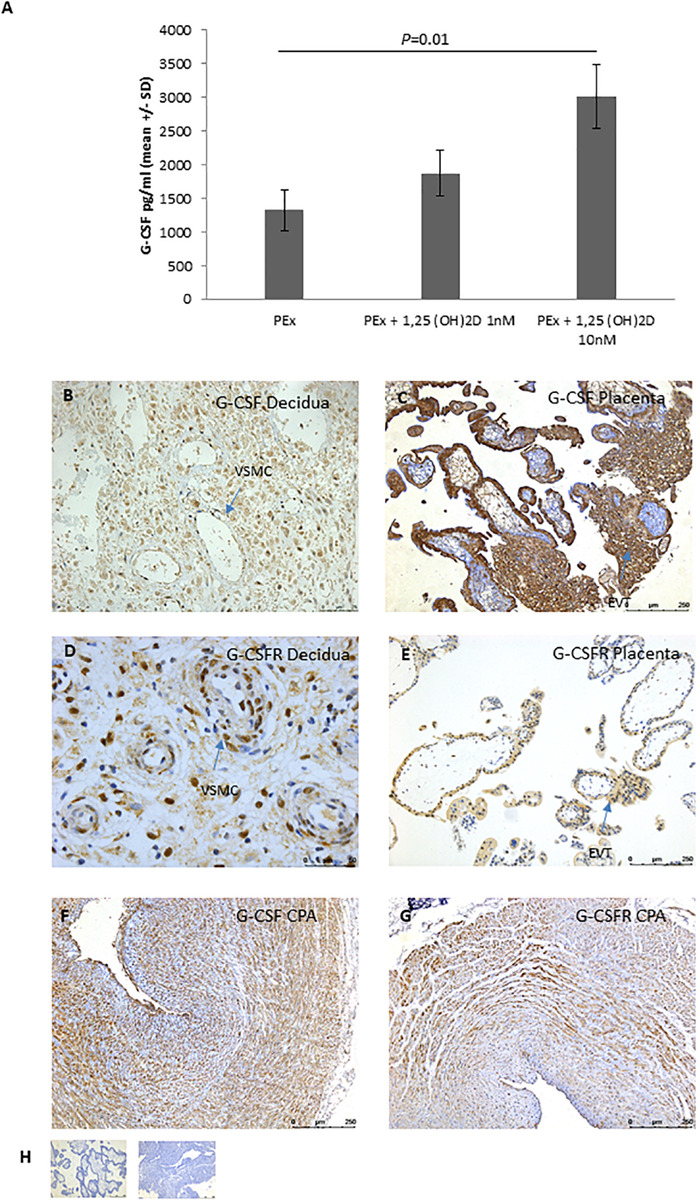
EVT expression of G-CSF. **(A)** Secretion of G-CSF by PEx after treatment with vitamin D; *n* = 5 each group. **(B–E)** Representative micrographs of immunohistological localization of G-CSF **(B,C)** and G-CSFR **(D,E)** in first trimester decidua basalis **(B,D)** and placenta **(C,E)**. **(F)** Representative micrograph of immunohistological localization of G-CSF to CPA. **(G)** Representative micrograph of immunohistological localization of G-CSFR to CPA. **(H)** Representative negative control with mouse IgG (placenta) or rabbit IgG (decidua). EVT, extravillous trophoblast; VSMC, vascular smooth muscle cell.

To determine the potential mechanism by which 1,25(OH)_2_D altered PEx CM so that is was able to induce VSMC disorganization in the CPA model; we performed a wide panel Luminex multiplex array. Treatment with 1,25(OH)_2_D did not alter the secretion profile of uNK cells or uNK cell/PEx co-cultures (direct and indirect) (Supplementary Table 1). Treatment with 1,25(OH)_2_D increased secretion of granulocyte colony stimulating factor (G-CSF) by PEx in a dose dependent manner (10 nM *P* = 0.01; Figure 2A and Supplementary Table 1).

### G-CSF and G-CSFR Are Expressed in First Trimester Placenta and Decidua

To determine the spatial distribution of G-CSF and its receptor, G-CSFR, IHC was performed on first trimester decidua and placental tissue. In first trimester placenta G-CSF and G-CSFR were highly expressed by cytotrophoblast, syncytiotrophoblast and EVT of the cell columns (Figure 2C,E). In the decidua, G-CSF was predominantly localized to invading EVT and decidual stromal cells (Figure 2B). G-CSFR was also positively expressed by invading EVT and decidual stromal cells as well as the VSMCs of the spiral arteries (Figure 2D). G-CSFR also immunolocalized to VSMCs of the CPA model (Figure 2F).

### G-CSF Induces VSMC Disorganization in the CPA Model

**FIGURE 3:**
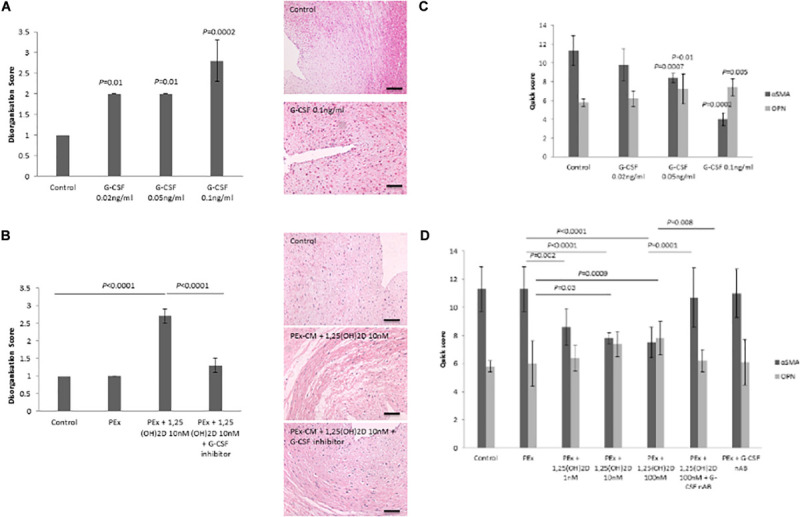
Effect of G-CSF on VSMC disorganization and phenotypic switching in the CPA model. **(A)** G-CSF induces VSMC disorganization in the CPA model of vascular remodeling. **(B)** Neutralization of G-CSF in vitamin D treated PEx CM abrogates the ability to induce VSMC disorganization. **(C)** G-CSF induces VSMC phenotypic from a contractile to synthetic phenotype as evidenced by reduced αSMA and increased osteopontin expression. **(D)** Neutralization of G-CSF in vitamin D treated PEx CM abrogates the ability to induce VSMC phenotypic switching. *N* = 5 each group. Scale bars = 50 μm.

To determine whether G-CSF induces VSMC disorganization and if so, whether increased G-CSF secretion underlies 1,25(OH)_2_D treated PEx CM induction of VSMC disorganization, the CPA model was used under the following treatment conditions (i) G-CSF alone, (ii) 1,25(OH)_2_D treated PEx CM, and (iii) 1,25(OH)_2_D treated PEx CM pre-treated with G-CSF neutralizing antibody inhibitor. G-CSF induced VSMC disorganization in a dose dependent manner (0.02 ng/ml *P* = 0.01, 0.05 ng/ml *P* = 0.01, and 0.01 ng/ml *P* = 0.0002; Figure 3A). Pre-treatment of 1,25(OH)_2_D treated PEx CM with G-CSF neutralizing antibody inhibited the ability of 1,25(OH)_2_D treated PEx CM to induce VSMC disorganization (*P* < 0.0001; Figure 3B).

Another feature of SpA remodeling is phenotypic switching of the VSMCs from a contractile to a synthetic phenotype, as characterized by a decrease in αSMA and an increase in osteopontin expression (Robson et al., 2019). In the CPA model, G-CSF induced VSMC phenotypic switching (αSMA *P* = 0.0002; OPN *P* = 0.005; Figure 3C). 1,25(OH)_2_D treated PEx CM induced VSMC phenotypic switching (αSMA *P* < 0.0001; OPN *P* = 0.0009; Figure 3D), an effect that was abrogated following pre-treatment with G-CSF neutralizing antibody (αSMA *P* = 0.0001; OPN *P* = 0.008; Figure 3D).

### G-CSF Induces VSMC Phenotypic Switching From a Contractile to Synthetic State

**FIGURE 4:**
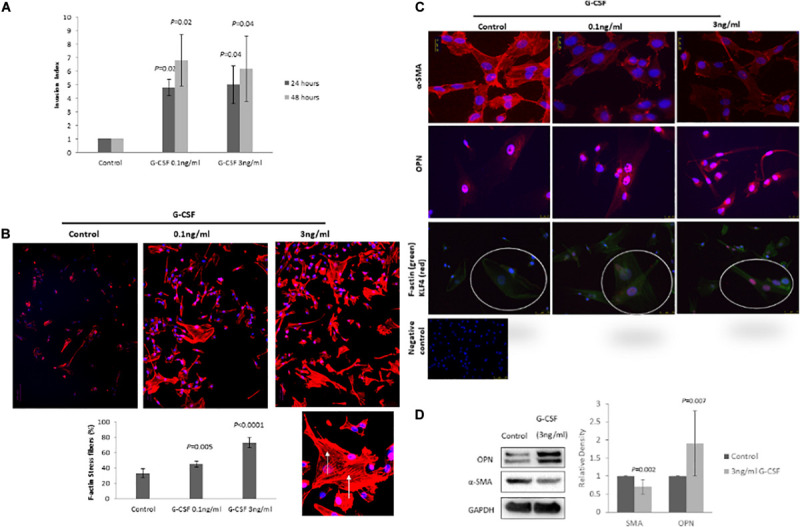
G-CSF induces VSMC phenotypic switching *in vitro*. **(A)** G-CSF induces VSMC invasion. **(B)** G-CSF increases the number of F-actin stress fibers in VMCs. Arrows denote F-actin stress fibers in a high power image. **(C)** Representative photomicrograph of IF staining for αSMA, osteopontin and KLF4 (red)/F-actin (green). High power inset of one cell double IF stained for KLF4 (red)/F-actin (green). IgG negative control. Note increased αSMA and KLF4, and reduced osteopontin as markers of VSMC phenotypic switching from a contractile to synthetic phenotype. *N* = 3 all groups. **(D)** Representative Western blots for αSMA, OPN, and GAPDH with corresponding quantitation for VSMCs treated with 3 ng/ml G-CSF for 24 h (*n* = 10).

Vascular smooth muscle cells show a remarkable plasticity and are not terminally differentiated, therefore they are able to switch from a “synthetic” to a “differentiated contractile” phenotype and vice versa in response to different environmental cues. To determine whether G-CSF has direct effects on VSMC phenotypic switching we measured expression of recognized VSMC phenotype markers and VSMC motile function, a synthetic phenotype feature (Owens, 2007). G-CSF stimulated VSMC invasion after 24 h (0.1 ng/ml *P* = 0.02 and 3 ng/ml *P* = 0.02) and 48 h (0.1 ng/ml *P* = 0.04 and 3 ng/ml *P* = 0.04) time-points (Figure 4A). G-CSF had no effect on VSCM proliferation (data not shown). In accordance with an increased motile phenotype a significant increase in F-actin stress fibers in VSMC following treatment with G-CSF was measured (0.1 ng/ml *P* = 0.005 and 3 ng/ml *P* < 0.0001; Figure 4B). In addition, G-CSF reduced expression of αSMA and increased osteopontin expression as shown by both immunofluorescence and Western blot analysis (Figure 4C,D), consistent with a more synthetic phenotype. G-CSF also induced nuclear expression of the transcription factor KLF4, the key transcription factor for mediating phenotypic switching from a contractile to synthetic phenotype (Figure 4C).

## Discussion

Remodeling of the uterine spiral arteries is one of the key maternal adaptations required for the establishment of a successful hemochorial pregnancy, with failure of this process leading to a number of serious complications of pregnancy, increasing both maternal and fetal mortality and morbidity. In epidemiological studies, maternal vitamin D deficiency has also been associated with a risk of developing pregnancy complications, although the etiology of this association is not well understood. This current study demonstrates that active 1,25 (OH)_2_D is able to induce placental explants (EVT) to mediate the initial stages of vascular remodeling, *via* a mechanism associated with G-CSF secretion. In addition, we demonstrate a direct role for G-CSF in VSMC phenotype switching from a contractile to synthetic state.

Spiral artery remodeling, key to hemochorial placentation, is primarily described in terms of its morphological features, swelling of the endothelium, rounding up and separation of VSMCs, EVT invasion into the vessel wall, VSMC phenotype switching, fibrinoid deposition, loss of VSMCs from the vessel wall *via* migration and then apoptosis (Pijnenborg et al., 2006; Bulmer et al., 2012). This results in relatively large bore, low resistance vessels which deliver large volumes of maternal blood to the placenta without pulsatile flow. The initial stages of remodeling occur in the absence of EVT, although this cell type is required for complete remodeling, in particular for maintaining the integrity of the vascular wall in fully remodeled vessels which have a complete endothelial lining over a wall of intramural EVT embedded in fibrinoid (Craven et al., 1998; Kam et al., 1999; Pijnenborg et al., 2006). Various leucocyte populations are associated with the SpA wall and were proposed to play roles in the initial stages of SpA remodeling (Smith et al., 2009). We have previously shown that uNK cells can induce VSMC rounding and separation, whilst EVT and EVT/uNK cell co-cultures have no effect on this aspect of SpA remodeling (Robson et al., 2012). Similar results were observed in the current study, although we used placental explants as a proxy for isolated EVT. Vitamin D has previously been shown to inhibit cytokine secretion by human uNK cells and trophoblast cells, although this has often been investigated within an inflammatory context (Evans et al., 2006; Barrera et al., 2012). However, in the current study neither 1,25(OH)_2_D or 25OHD altered the ability of uNK cells to induce VSMC disorganization in the CPA model of SpA remodeling, and did not alter cytokine secretion. Consistent with this, RNA-sequence analysis of 1,25(OH)_2_D-treated uNK cells identified no significant difference in cytokine, vessel remodeling or angiogenic transcript expression comparative to paired 1,25(OH)_2_D-treated pNK cells following immune activation (Tamblyn et al., 2019).

We have previously shown that there is an inhibition of cytokine and angiogenic growth factor secretion in uNK cells and EVT co-culture, either in direct contact or separated by a transwell (Lash et al., 2011). In addition, conditioned medium from these co-cultures did not induce VSMC disorganization or phenotypic switching, likely due to the decrease in cytokines and angiogenic growth factors (Robson et al., 2012, 2019). Similar results were found in the current study, and addition of 1,25(OH)_2_D did not modify conditioned media effects upon VSMC organization in the CPA model.

Our previous studies demonstrate that while EVT do not appear to play a role in VSMC disorganization they are able to induce VSMC phenotype switching (Robson et al., 2012, 2019). Others have shown that EVT derived exosomes, CXCL10 and long non-coding RNA MEG3 may also play roles in increasing VSMC motility and phenotypic switching to a more synthetic phenotype, as observed *ex vivo* (Wallace et al., 2013; Salomon et al., 2014; Robson et al., 2019; Tao et al., 2019). This is a complex process requiring the co-ordinated effects of several different cell types, and likely mediated by more than one factor. In the current study we used placental explants as a source of EVT and, in agreement with our previous work, they did not induce VSMC disorganization. However, after culture with 1,25(OH)_2_D, but not 25OHD, placental explant conditioned medium induced VSMC disorganization in the CPA model. A wide screen multiplex Luminex panel of cytokines, chemokines, and angiogenic growth factors demonstrated that 1,25(OH)_2_D increased secretion of G-CSF by the placental explants, but no other differences were observed in any culture condition. Few other studies have investigated secretion of G-CSF after vitamin D treatment, despite its proposed immunomodulatory role. In agreement with the current study, treatment with vitamin D increased G-CSF secretion by peripheral blood NK cells (Ota et al., 2014). However, to the best of our knowledge this is the first study to demonstrate that active vitamin D stimulates G-CSF secretion by placental explants or trophoblast cells.

Granulocyte-colony stimulating factor is a 30 kDa glycoprotein that is expressed by a range of cell types and is predominantly involved in release of hemopoietic progenitor cells from the bone marrow and their differentiation into granulocytes within the circulation (Yoshioka et al., 2006; Akihama et al., 2007). More recent evidence also suggests that it can recruit endothelial progenitor cells to sites of injury (Kong et al., 2004). Therapeutically it is often used as an adjuvant therapy after chemotherapy in patients who experience myelosuppression. However, it has been noted that one side effect of this adjuvant treatment is an increase in tumor angiogenesis in some, but not all, patients (Shojaei et al., 2009; Voloshin et al., 2011; Aliper et al., 2014). Several studies have demonstrated the pro-angiogenic effect of G-CSF, likely *via* the recruitment of endothelial progenitor cells, but this effect appears to be site and dose specific, and interaction with the local microenvironment also plays a significant role in this effect (Sato et al., 2008; Cai et al., 2017; Li et al., 2019). Very few studies have investigated the effects of G-CSF on VSMC biology, with differing results. In a rat carotid artery injury model VSMC expression of G-CSF is increased (Chen et al., 2005). In addition, G-CSF induced migration of human coronary artery VSMCs (*in vitro*) *via* a mechanism associated with Rac1 signaling (Chen et al., 2005). These data support a role for G-CSF in phenotypic switching of VSMC to a more synthetic phenotype, though other markers were not investigated. In another rat carotid injury model, G-CSF appeared to stimulate both VSMC proliferation and differentiation to a more contractile phenotype (Rinaldi et al., 2016). Increased proliferation is a feature of VSMCs in a more synthetic state, while increased expression of more contractile phenotype markers was only prevalent by 30 days post treatment initiation. Therefore, these results may reflect alterations in function based on length of exposure and expression levels of other factors within the microenvironment. In our *ex vivo* vessel remodeling model and *in vitro* VSMC culture experiments we clearly demonstrate that G-CSF induces VSMC phenotypic switching to a more synthetic phenotype. In the *ex vivo* model the effect on osteopontin is subtle, but taken together with the opposite shift in αSMA levels, and the more robust changes observed in VSMCs *in vitro*, supports this hypothesis. In addition, to the direct effects of G-CSF in both the *ex vivo* and *in vitro* models, inhibition of G-CSF attenuated the effect of 1,25(OH)_2_D treated PEx in the *ex vivo* model suggesting that G-CSF was responsible for the observed effects and not any residual 1,25(OH)_2_D in the conditioned medium. However, more research in different vascular beds and in scenarios of physiological remodeling, as seen in early pregnancy, and pathological remodeling, as seen after vascular injury, are required to fully understand the role of G-CSF in these processes.

Vitamin D deficiency has been associated with a number of serious complications of pregnancy, and we originally hypothesized that it may alter the ability of different cells to mediate SpA remodeling, a key step in the establishment of a successful pregnancy. Our data suggests that vitamin D can enhance the ability of EVT to induce SpA remodeling *via* an increase in G-CSF secretion. In addition, vitamin D has previously been shown to stimulate EVT invasion (Chan et al., 2015). Therefore, in a scenario of vitamin D deficiency there would be reduced EVT invasion and reduced secretion of G-CSF both contributing to a reduction in SpA remodeling, and contributing to the etiology of complications of pregnancy. In the current study we were only able to demonstrate G-CSF effects with 1,25(OH)_2_D and not 25(OH)D, the main circulating marker of vitamin D status. This may simply reflect the relatively low levels of 1,25(OH)_2_D synthesis from added 25(OH)D in the explant model used for these experiments. However, in future studies it will be interesting to assess the effects of higher levels of 25(OH)D for longer incubation periods. A few studies have investigated the association of serum G-CSF with pre-eclampsia, one found increased, one decreased and one unaltered levels either at the time of delivery or in the second trimester, therefore it is not clear whether circulating levels contribute to complications of pregnancy (Matsubara et al., 1999; Polliotti et al., 2003; Chedraui et al., 2009). However, changes in serum G-CSF may not reflect local concentrations within the maternal-fetal interface and its paracrine/autocrine effects are likely to be more important for a contribution to the etiology of complications of pregnancy. Pre-conception administration of G-CSF may also be beneficial for a subset of women with recurrent pregnancy loss or recurrent implantation failure, although the mechanism of action is not yet fully understood (Santjohanser et al., 2013; Eapen et al., 2019; Kamath et al., 2020; Rocha et al., 2020).

The current study relies on the use of *in vitro* tissue and cellular models, and as such it is not known if the results can be fully extrapolated into the *in vivo* situation. The vessel model makes use of the easily accessible chorionic plate arteries from term placenta. These vessels contain several layers of VSMCs and can be obtained in a non-invasive manner without requirement for unnecessary patient procedures. The model has been validated against sections of spiral arteries dissected from non-pregnant myometrium after hysterectomy (Robson et al., 2012). However, this model does only lend itself to semiquantitative analysis. To further show a role for G-CSF in VSMC phenotypic switching we used a commercial source of VSMCs. Further studies should include other cellular models as well as appropriate animal models.

In conclusion, in the current study we demonstrate that vitamin D can enhance the ability of EVT to induce vascular remodeling, *via* a mechanism associated with increased secretion of G-CSF that in turn is able to increase VSMC disorganization and phenotypic switching in both an *ex vivo* vascular remodeling model and *in vitro* VSMC cultures. This is one of the first studies showing a direct effect of G-CSF on VSMC biology, although one previous study has also reported increased VSMC migration in response to G-CSF. The clinical relevance of these results is still to be determined as it appears that G-CSF may have differential effects depending on dose and vascular bed. However, it may be an interesting potential therapeutic target for facilitating physiological vascular remodeling for the prevention of adverse obstetric outcomes.

## Data Availability Statement

The raw data supporting the conclusions of this article will be made available by the authors, without undue reservation.

## Ethics Statement

The studies involving human participants were reviewed and approved by Guangzhou Women and Children’s Medical Center Ethical Review Board. The patients/participants provided their written informed consent to participate in this study.

## Author Contributions

JZ and PW conceived the study and performed the experiments and data analysis. DC, FN, and QL performed the experiments. XQ, JT, MH, GL, and MK conceived the study, obtained funding, critically assessed the data, and aided in interpretation. GL wrote the manuscript. All authors edited the manuscript and approved its final version.

## Conflict of Interest

The authors declare that the research was conducted in the absence of any commercial or financial relationships that could be construed as a potential conflict of interest.
