# Supplementary material for: Vitamin D Promotes Trophoblast Cell Induced Separation of Vascular Smooth Muscle Cells in Vascular Remodeling via Induction of G-CSF
Source: Front Cell Dev Biol. 2020 Dec 22;8:601043. doi: 10.3389/fcell.2020.601043 (PMC7783206; doi:10.3389/fcell.2020.601043)
Supplement: Supplementary file 1 [file Table_1.DOCX]

Supplementary Table 1: Concentration of cytokines, chemokines and angiogenic growth factors secreted by uNK cells, placental explants or both in co-culture with or without addition of 1,25(OH)_2_D as determined by Luminex multiplex assays (pg/ml; mean ± SD; n=5)

|  | **uNK** | **uNK + 1,25(OH)_2_D 1nM** | **uNK + 1,25(OH)_2_D 10nM** | **PEx** | **PEx + 1,25(OH)_2_D 1nM** | **PEx + 1,25(OH)_2_D 10nM** | **uNK/PEx-dir** | **uNK/PEx-dir + 1,25(OH)_2_D 1nM** | **uNK/PEx-dir + 1,25(OH)_2_D 10nM** | **uNK/PEx-indir** | **uNK/PEx-indir + 1,25(OH)_2_D 1nM** | **uNK/PEx-indir + 1,25(OH)_2_D 10nM** |
| --- | --- | --- | --- | --- | --- | --- | --- | --- | --- | --- | --- | --- |
| **Ang2** | 847.9 ± 552.5 | 258.6 ± 53.7 | 284.3 ± 74.3 | 188501.6 ± 522440.4 | 165441.8 ± 43441.0 | 186522.6 ± 60751.4 | 168526.7 ± 62310.1 | 147921.7 ± 49393.6 | 188355.6 ± 56122.4 | 186779.5 ± 62457.1 | 142924.5 ± 49340.9 | 171646.0 ± 54863.5 |
| **EGF** | 1.0 ± 0.1 | 1.0 ± 0.1 | 1.1 ± 0.2 | 1.3 ± 0.04 | 1.2 ± 0.02 | 1.2 ± 0.02 | 1.0 ± 0.03 | 1.0 ± 0.04 | 1.0 ± 0.1 | 1.0 ± 0.1 | 1.0 ± 0.1 | 1.0 ± 0.1 |
| **FGF-2** | 170.6 ± 81.2 | 168.0 ± 75.6 | 150.9 ± 58.9 | 224.8 ± 27.8 | 220.7 ± 29.0 | 211.5 ± 25.6 | 318.3 ± 69.3 | 245.6 ± 47.3 | 263.5 ± 53.7 | 332.6 ± 53.0 | 297.3 ± 60.5 | 288.9 ± 60.0 |
| **G-CSF** | 129.8 ± 56.7 | 151.5 ± 67.8 | 163.6 ± 64.3 | 1327.3 ± 306.2 | 1875.8 ± 333.9 | ***3012.1 ± 470.8*** | 3680.7 ± 1561.1 | 1650.2 ± 326.7 | 2698.2 ± 477.4 | 3377.3 ± 557.6 | 2737.9 ± 1099.3 | 3053.1 ± 1418.1 |
| **GM-CSF** | 441.5 ± 438.4 | 403.5 ± 383.9 | 348.7 ± 337.2 | 943.0 ± 483.2 | 1358.6 ± 644.2 | 880.3 ± 255.2 | 1389.3 ± 465.1 | 1000.1 ± 288.4 | 1134.3 ± 341.7 | 1214.4 ± 375.3 | 1663.8 ± 545.2 | 1045.5 ± 453.4 |
| **GRO-α** | 649.4 ± 207.6 | 590.8 ± 190.7 | 528.7 ± 179.2 | 2496.9 ± 767.6 | 4958 ± 2011.5 | 5961.5 ± 2336.6 | 3811.7 ± 598.9 | 4774.4 ± 1140.1 | 10296.5 ± 3716.0 | 7659.5 ± 1043.8 | 5968.6 ± 2852.5 | 5961.5 ± 7465.0 |
| **IFN-γ** | 0.6 ± 0.04 | 0.7 ± 0.1 | 0.6 ± 0.1 | 1.9 ± 0.4 | 2.0 ± 0.3 | 1.9 ± 0.1 | 2.4 ± 0.6 | 1.7 ± 0.2 | 1.8 ± 0.2 | 3.3 ± 1.0 | 2.5 ± 0.7 | 2.1 ± 0.5 |
| **IL-1β** | 1.9 ± 0.4 | 1.9 ± 0.3 | 1.6 ± 0.3 | 0.4 ± 0.1 | 0.5 ± 0.1 | 0.6 ± 0.2 | 1.0 ± 0.2 | 1.3 ± 0.2 | 1.0 ± 0.1 | 1.4 ± 0.2 | 1.6 ± 0.3 | 1.4 ± 0.3 |
| **IL-1Rα** | 3.0 ± 1.0 | 2.1 ± 0.4 | 2.8 ± 1.0 | 5.0 ± 1.3 | 7.7 ± 3.7 | 3.9 ± 1.5 | 5.2 ± 1.9 | 6.0 ± 2.2 | 5.4 ± 1.4 | 3.4 ± 0.9 | 5.5 ± 2.0 | 3.7 ± 0.8 |
| **IL-4** | 7.7 ± 2.3 | 4.9 ± 1.5 | 4.1 ± 1.1 | 3.4 ± 0.8 | 2.9 ± 1.1 | 1.3 ± 0.2 | 8.5 ± 1.8 | 9.7 ± 3.3 | 9.2 ± 4.5 | 3.2 ± 1.2 | 3.4 ± 1.3 | 5.3 ± 1.5 |
| **IL-6** | 226.3 ±130.6 | 151.3 ± 125.2 | 126.5 ± 100.4 | 5907.1 ± 820.5 | 5909.3 ± 478.7 | 6278.9 ± 344.3 | 6121.3 ± 528.4 | 6826.8 ± 482.6 | 6658.6 ± 239.8 | 6665.0 ± 458.2 | 6695.4 ± 341.8 | 6288.0 ± 704.6 |
| **IP-10** | 90.2 ± 27.1 | 91.6 ± 29.7 | 72.1 ± 21.8 | 193.5 ± 49.2 | 197.2 ± 35.1 | 181.6 ± 46.3 | 401.7 ± 120.6 | 355.5 ± 71.0 | 391.0 ± 96.7 | 396.1 ± 109.0 | 356.3 ± 84.7 | 356.4 ± 67.1 |
| **MCP-1** | 494.2 ± 183.5 | 469.3 ± 160.6 | 420.1 ± 133.6 | 2362.6 ± 320.7 | 2057.1 ± 256.8 | 2035.5 ± 318.9 | 2119.7 ± 309.6 | 2120.8 ± 315.3 | 1960.8 ± 372.3 | 2407.5 ± 241.2 | 2309.0 ± 217.6 | 2091.1 ± 341.4 |
| **MCP-3** | 12.4 ± 2.7 | 11.9 ± 1.5 | 9.7 ± 0.9 | 48.4 ± 23.9 | 39.2 ± 15.9 | 62.2 ± 34.0 | 107.1 ± 59.6 | 106.0 ± 63.8 | 131.8 ± 76.0 | 107.1 ± 57.4 | 77.6 ± 31.1 | 87.6 ± 40.0 |
| **MDC** | 17.3 ± 5.6 | 15.0 ± 4.7 | 9.6 ± 2.5 | 9.2 ± 3.0 | 12.1 ± 3.2 | 19.5 ± 6.7 | 39.7 ± 8.6 | 48.2 ± 11.6 | 46.5 ± 11.4 | 58.5 ± 12.4 | 68.3 ± 17.9 | 65.8 ± 21.6 |
| **MIP-1α** | 311.6 ± 94.4 | 293.5 ± 81.4 | 262.5 ± 81.8 | 615.2 ± 221.7 | 411.3 ± 108.4 | 559.8 ± 180.6 | 533.0 ± 149.8 | 447.3 ± 96.9 | 458.9 ± 118.4 | 739.5 ± 178.7 | 654.0 ± 129.7 | 664.5 ± 177.1 |
| **MIP-1β** | 30.5 ± 7.7 | 29.8 ± 8.9 | 27.6 ± 9.4 | 186.7 ± 63.7 | 151.3 ± 54.5 | 175.6 ± 82.1 | 172.9 ± 65.4 | 142.0 ± 35.7 | 147.2 ± 59.5 | 140.9 ± 51.5 | 129.1 ± 32 | 140.6 ± 43.2 |
| **PDGF-AA** | 0.6 ± 0.2 | 0.4 ± 0.2 | 1.4 ± 0.8 | 41.0 ± 6.3 | 48.7 ± 4.2 | 40.5 ± 7.5 | 40.0 ± 6.3 | 39.6 ± 5.2 | 42.6 ± 7.3 | 28.4 ± 7.0 | 36.4 ± 6.1 | 37.8 ± 11.2 |
| **PDGF-BB** | 1.3 ± 0.6 | 0.1 ± 0.0 | 14.4 ± 12.5 | 93.1 ± 24.9 | 86.3 ± 14.4 | 81.0 ± 16.2 | 83.5 ± 19.6 | 86.8 ± 25.9 | 103.9 ± 29.3 | 61.9 ± 22.2 | 64.7 ± 26.1 | 79.3 ± 30.9 |
| **PlGF** | 7.3 ± 6.0 | 1.2 ± 0.5 | 1.1 ± 0.6 | 209.2 ± 63.3 | 184.0 ± 47.9 | 131.1 ± 30.5 | 266.9 ± 84.0 | 377.8 ± 138.9 | 127.7 ± 20.8 | 104.4 ± 22.3 | 178.5 ± 78.4 | 189.9 ± 76.3 |
| **RANTES** | 25.6 ± 7.4 | 25.7 ± 8.7 | 25.5 ± 11.1 | 131.3 ± 26.5 | 123.7 ± 24.4 | 120.0 ± 24.8 | 133.8 ± 28.8 | 90.1 ± 20.6 | 108.3 ± 23.2 | 145.0 ± 24.2 | 110.9 ± 23.5 | 124.0 ±34.4 |
| **TNF-α** | 10.0 ± 4.2 | 8.5 ± 3.0 | 7.0 ± 1.9 | 121.5 ± 23.0 | 90.9 ± 18.7 | 93.7 ± 13.2 | 102.2 ± 16.7 | 97.7 ± 9.4 | 91.1 ± 10.8 | 128.2 ± 13.4 | 98.5 ± 8.1 | 108.6 ± 12.1 |
| **VEGF-A** | 2.5 ± 0.9 | 4.5 ± 1.4 | 5.0 ± 0.9 | 17.5 ± 4.2 | 19.2 ± 1.8 | 22.0 ± 1.6 | 19.4 ± 2.4 | 21.5 ± 3.2 | 22.3 ± 4.1 | 24.0 ± 3.3 | 21.6 ± 4.0 | 18.4 ± 2.1 |
